# Enhancing the ophthalmic AI assessment with a fundus image quality classifier using local and global attention mechanisms

**DOI:** 10.3389/fmed.2024.1418048

**Published:** 2024-08-07

**Authors:** Shengzhan Wang, Wenyue Shen, Zhiyuan Gao, Xiaoyu Jiang, Yaqi Wang, Yunxiang Li, Xiaoyu Ma, Wenhao Wang, Shuanghua Xin, Weina Ren, Kai Jin, Juan Ye

**Affiliations:** ^1^The Affiliated People’s Hospital of Ningbo University, Ningbo, Zhejiang, China; ^2^Eye Center, School of Medicine, The Second Affiliated Hospital, Zhejiang University, Hangzhou, Zhejiang, China; ^3^College of Control Science and Engineering, Zhejiang University, Hangzhou, China; ^4^College of Media, Communication University of Zhejiang, Hangzhou, China; ^5^College of Computer Science and Technology, Hangzhou Dianzi University, Hangzhou, China; ^6^Institute of Intelligent Media, Communication University of Zhejiang, Hangzhou, China

**Keywords:** fundus photography, attention mechanism, image quality assessment, spatial information, multiscale feature extraction

## Abstract

**Background:**

The assessment of image quality (IQA) plays a pivotal role in the realm of image-based computer-aided diagnosis techniques, with fundus imaging standing as the primary method for the screening and diagnosis of ophthalmic diseases. Conventional studies on fundus IQA tend to rely on simplistic datasets for evaluation, predominantly focusing on either local or global information, rather than a synthesis of both. Moreover, the interpretability of these studies often lacks compelling evidence. In order to address these issues, this study introduces the Local and Global Attention Aggregated Deep Neural Network (LGAANet), an innovative approach that integrates both local and global information for enhanced analysis.

**Methods:**

The LGAANet was developed and validated using a Multi-Source Heterogeneous Fundus (MSHF) database, encompassing a diverse collection of images. This dataset includes 802 color fundus photography (CFP) images (302 from portable cameras), and 500 ultrawide-field (UWF) images from 904 patients with diabetic retinopathy (DR) and glaucoma, as well as healthy individuals. The assessment of image quality was meticulously carried out by a trio of ophthalmologists, leveraging the human visual system as a benchmark. Furthermore, the model employs attention mechanisms and saliency maps to bolster its interpretability.

**Results:**

In testing with the CFP dataset, LGAANet demonstrated remarkable accuracy in three critical dimensions of image quality (illumination, clarity and contrast based on the characteristics of human visual system, and indicates the potential aspects to improve the image quality), recording scores of 0.947, 0.924, and 0.947, respectively. Similarly, when applied to the UWF dataset, the model achieved accuracies of 0.889, 0.913, and 0.923, respectively. These results underscore the efficacy of LGAANet in distinguishing between varying degrees of image quality with high precision.

**Conclusion:**

To our knowledge, LGAANet represents the inaugural algorithm trained on an MSHF dataset specifically for fundus IQA, marking a significant milestone in the advancement of computer-aided diagnosis in ophthalmology. This research significantly contributes to the field, offering a novel methodology for the assessment and interpretation of fundus images in the detection and diagnosis of ocular diseases.

## Introduction

Fundus photography stands as a cornerstone in the diagnosis of diabetic retinopathy (DR), glaucoma, age-related macular degeneration (AMD), among various ocular disorders (1). With the advent of artificial intelligence (AI), the automation of disease screening through fundus imaging has emerged as a focal area of research and clinical application (2). Several algorithms have been explored, with a notable number being translated into clinical settings (3–5). The quality of fundus images is critical to the diagnostic accuracy of these models, necessitating a robust Image Quality Assessment (IQA) for automated systems.

Manual IQA, though reliable, places a significant burden on medical professionals which requires direct assessment of images to ensure pathological structures are discernibly visible. Conversely, automated IQA methods offer a less labor-intensive alternative, utilizing algorithms to evaluate image quality. These methods range from structure-analysis-based to generic image-statistics approaches (6). In the era of deep learning, innovations in IQA have significantly benefited from the advanced feature-extraction capabilities of convolutional neural networks (CNNs) (7–9), employing strategies such as hallucinated reference generation and distortion identification to enhance quality prediction and feature weighting through visual saliency (10). DeepFundus, a deep learning-based fundus image classifier, addresses the data quality gap in medical AI by offering automated, multidimensional image sorting, significantly enhancing model performance across various retinopathies and supporting a data-driven paradigm for the entire medical AI lifecycle (11).

Despite these advancements, challenges persist, particularly in the generalizability of algorithms across diverse imaging conditions and the integration of both local and global information critical for comprehensive quality assessment. Furthermore, the interpretability of deep learning models in this context remains uncertain. In order to fill these gaps, this study introduces the Local and Global Attention Aggregated Deep Neural Network (LGAANet), designed to leverage both local and global information in assessing the quality of fundus images. Most existing IQA datasets are single-center collections that overlook variations in imaging devices, eye conditions, and imaging environments. Our approach involves training on a multi-source heterogeneous fundus (MSHF) database (12), encompassing a broad spectrum of normal and pathological images captured through various imaging modalities, to enhance the model’s generalizability and interpretability. This database was selected due to its diverse and representative nature, which allows for robust validation of the LGAANet model across various imaging conditions and sources.

## Materials and methods

**FIGURE 1:**
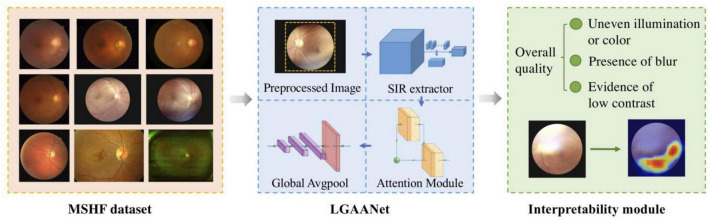
An overview of the study approach and methodology. The multi-source heterogeneous fundus (MSHF) dataset is collected, and then serves as an input to train the local and global attention aggregated deep neural network (LGAANet). The output is the image quality of each image based on three metrics, and a heap map is created to show the interpretability.

An overview of the study approach and methodology is presented in Figure 1. Our MSHF dataset consisted of various sub-databases collected from different devices and exhibited diverse appearance patterns. The dataset comprises 802 color fundus photography (CFP) images (302 from portable fundus cameras) and 500 ultrawide-field (UWF) images. These images originate from 904 patients, encompassing DR and glaucoma patients, in addition to normal individuals. Such samples collected via various domains are capable of providing more diversity during training of CNNs, which is beneficial for improving the generalization ability of models. Three critical dimensions of image quality: the illumination, clarity and contrast are selected based on the characteristics of human visual system, and indicates the potential aspects to improve the image quality. In order to validate the performance of our approach, we used an external dataset and noise dataset. A detailed description of each stage follows.

### The spatial-information-retained multi-scale feature extractor

Multi-scale features and spatial attention mechanisms have shown potential for quality prediction (13–19). However, existing multi-scale-feature-incorporated quality-prediction studies tend to leverage Multi-Level Spatially Pooled (MLSP) strategy to aggregate features from various scales, i.e., using Global Average Pooling (GAP) to extract the multi-dimensional activations into a one-dimensional vector and concatenate vectors from various scales. The MLSP method yields one-dimensional vectors and inevitably leaves out much spatial information. Therefore, it is challenging to integrate spatial attention mechanisms into the one-dimensional feature.

In order to improve prediction accuracy and combine both multi-scale features and spatial mechanisms into our quality prediction model, we included a spatial-information-retained (SIR) multi-scale feature extractor to combine both local and global quality-aware features through an attention-incorporated perspective.

Specifically, let *X* denote the input image with size [3, *H*, *W*], and denote the multi-scale feature (Scale#1 to Scale #3) extracted from ResNet50 as:


(1) $$s_{i}=f\,\left(X|S\,t\,a\,g\,e_{i}\right),i\in \left\{1,2,3\right\}$$

Where *f*(⋅|*Stage*_*i*_) denotes the activations extracted from the last convolutional layer of ResNet50 in Stage#*i*. The *s_i_* is rescaled channel-wise via a convolutional layer with kernel size 1x1 and followed by a batch-normalization and a RELU layer, i.e., s=′ig(si|TIi,Oi1,0), in which $g\,\left(\text{⋅}|T_{C_{i\,n},C_{o\,u\,t}}^{k,p}\right)$ denotes the convolutional unit mentioned above with kernel size, padding, input channel size *C*_*in*_, and output channel size *C*_*out*_. In the architecture of ResNet50, [*I*_1_, *I*_2_, *I*_3_] = [256, 512, 1024], and we set [*O*_1_, *O*_2_, *O*_3_] = [16, 32, 64] to prevent the channel size after concatenation from being too large. Therefore, the size of s,′1s,′2s,′3 is [16, *W*/4, *H*/4], [32, *W*/8, *H*/8], [64, *W*/16, *H*/16], respectively.

In order to maintain the detailed spatial information of features extracted from each scale and simultaneously rescale them to coordinate with features extracted from the last Stage of ResNet50 (i.e., Stage#4 with spatial size [*W*/32, *H*/32]), the s,′1s,′2s,′3 are non-overlapped and spatially split into several chunks with spatial size [*W*/32, *H*/32], i.e.,:


(2) c⁢h⁢u⁢n⁢ki=s⁢p⁢l⁢i⁢t⁢(s′i)=[c1,1(i)⁢⋯⁢c1,ki(i)⋮⁢⋱⁢⋮cki,1(i)⁢⋯⁢cki,ki(i)]

Where *chunk*_*i*_ denotes the set of chunks after spatial split from s′i, and each of the chunks is denoted as $c_{m,n}^{\left(i\right)}$ (*m* and *n* denote the spatial index of the chunk) with a channel size coordinated with s′i and a spatial size of [*W*/32, *H*/32]. In addition, *k*_1_ = 64, *k*_2_ = 16, *k*_3_ = 4.

As for each *chunk*_*i*_, its elements are concatenated channel-wise by,


(3) s=″iconcat({cm,n(i)|m∈ki,n∈ki},dimchannel_wise)

After this, the size of s,″1s,″2s,″3 is [16*64, *W*/32, *H*/32], [32*16, *W*/32, *H*/32], [64*4, *W*/32, *H*/32]. Finally, s,″1s,″2s,″3 and the activations extracted via *f*(⋅|*Stage*_4_) are fed into $g\,\left(\text{⋅}|T_{C_{i\,n},128}^{1,0}\right)$ and yield 4 multi-dimensional features with the same size, representing both local and global information. Channel-wise concatenation is then employed to obtain a local spatial-information-retained multi-scale feature with size [128*4, *W*/32, *H*/32].

**FIGURE 2:**
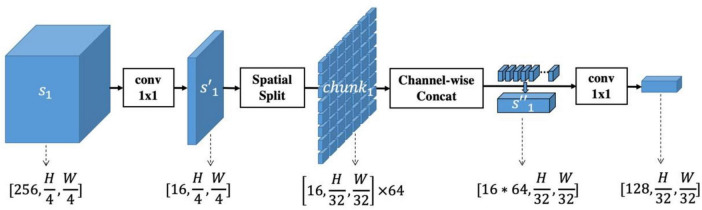
Illustration of spatial-information-retained (SIR) multi-scale feature extraction. The activations extracted from Stage#1 of ResNet50, denoted as *s_1_*, are first rescaled into s′1 by a convolutional layer with kernel size 1x1. Then s′1 is spatially split into multiple chunks whose spatial size is coordinated with the features extracted from Stage#4 of ResNet50. The chunks are concatenated into s″1 and rescaled to a size of [128, *H*/32, *W*/32]. In this way, the spatial information of multi-scale features is retained while the feature size within each scale is consistent.

The above-described spatial-information-retained multi-scale feature extraction is also illustrated in Figure 2, taking Stage#1 as an example, and the pseudocode is listed in Table 1.

**TABLE 1:** Pseudocode of spatial-information-retained multi-scale feature extractor.

Let *X* denote the input image
Step1. Extract multi-scale feature *s_i_*, *i* = {1,2,3} from ResNet50 according to Equation 1
Step2. For each scale *i*:
Rescale *s_i_* via s=′ig(si|TIi,Oi1,0) channel-wise
Spatially split *s_i_* into *chunk*_*i*_ according to Equation 2
Concatenate elements in *chunk*_*i*_ channel-wise according to Equation 3 and obtain s″i
Rescale s″i channel-wise via $g\,\left(\text{⋅}|T_{C_{i\,n},128}^{1,0}\right)$ according to Equations 4, 5 and obtain *ft*_*i*_
End
Step3. Get *ft*_4_ by feeding *f*(X|*Stage*_4_) into $g\,\left(\text{⋅}|T_{C_{i\,n},128}^{1,0}\right)$
Step4. Concatenate {*ft*_*i*_|*i*ϵ[1,4]} channel-wise and obtain the final spatial-information-retained multi-scale feature

### LGAANet

**FIGURE 3:**
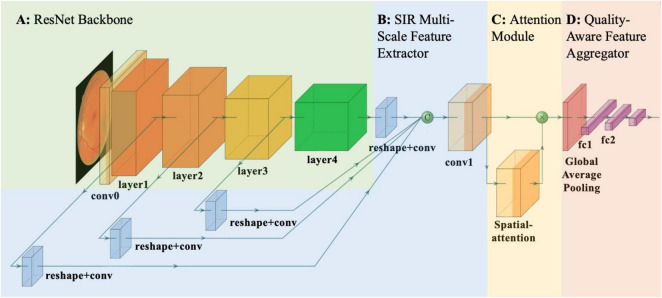
Overall pipeline of proposed local and global attention aggregated deep neural network (LGAANet) for quality prediction. **(A)** ResNet50 structure. **(B)** Spatial-information-retained (SIR) multi-scale feature extractor illustrated in Figure 2 and Section Methods-D. The green sphere labeled “C” denotes channel-wise concatenation of SIR features extracted at each scale. **(C)** The attention module is leveraged to learn the spatial weighting strategies and multiplied elemental-wise with the SIR multi-scale feature. **(D)** The global average pooling layer is incorporated and followed by several fully connected layers to aggregate the quality prediction.

Based on the proposed SIR multi-scale feature extractor, we developed the LGAANet, as shown in Figure 3. Our LGAANet is comprised of a ResNet50-based SIR multi-scale feature extractor *f*(⋅;θ), an attention module *Att*(⋅;γ), and a feature-aggregation module *g*(⋅;δ). Let *X* denote the input image; the final quality prediction $\hat{q}$ is obtained via,


(4) $$\hat{q}=g\,\left(f\,\left(X;\theta \right)\times a\,t\,t\,\left(f\,\left(X;\theta \right);\gamma \right);\delta \right)$$

Since the quality label *q* is binary, the loss to be optimized, denoted as *L*, is calculated by,


(5) $$L=B\,C\,E\,\left(S\,i\,g\,m\,o\,i\,d\,\left(\hat{q}\right),q\right)$$

Where *Sigmoid*(⋅) denotes the Sigmoid layer and *BCE*(⋅) denotes the binary cross-entropy.

The attention mechanism could be implemented via various CNN architectures. Here spatial attention [denoted as BaseLine (BL) + SpatialAtt + MultiScale (MS)] and self-attention (denoted as BL+SelfAtt+MS) are leveraged to learn the spatial weighting strategy for multi-scale quality-aware features. The spatial attention is implemented by several stacks of convolutional-batch normalization-RELU units while the self-attention is following (20). Also, we constructed a multi-scale excluded and attention-incorporated CNN framework for the ablation study, denoted as BL+SpatialAtt.

For the sake of comparison, we considered the BL in the performance comparison, in which the feature extracted from ResNet50 was directly fed into a GAP followed by stacks of the fully-connected layer. The MASK-incorporated model is also involved (denoted as BL+MASK) and has an overall pipeline similar to the BL, but the extracted features are multiplied elemental-wise with the MASK signal before being fed into the GAP layer.

Network hyperparameters: the minibatch size is 8, and the learning rate is 1e-3. The optimizer is Adam, and the weight-decay is 5e-4. The ratio of the learning rate of the ResNet model parameters to the subsequent newly added layer is 1:10; that is, the learning rate of the newly added layer is 1e-3, and of the ResNet layer is 1e-4. The training process traverses the training data in the database 20 times, which means the epoch = 20, and the highest test accuracy is selected as the final result. The division of training-test samples is randomly generated (a total of two, namely round = 2). The image index being used for training/testing is in the supplementary files teIdx01.mat (first test index), trIdx01.mat (first-time training index), teIdx02.mat (second test index), trIdx02.mat (second training index). The host configuration is i7-8700 CPU @3.2GHz & 32GB RAM + GTX1080@8GB.

To facilitate the development of deep learning models using the MSHF dataset, it was manually segmented into an 80% training set and a 20% test set. The training set facilitated model learning, while the test set served for performance evaluation. There was no overlap between these two sets, ensuring a fair distribution of image variety. Each set maintained an approximately equal proportion of high- and low-quality images.

### Statistical methods

For statistical validation, we employed a stratified 5-fold cross-validation technique to ensure that each subset of data was representative of the overall distribution, thus mitigating any potential bias due to imbalanced data. This method involved dividing the data into 5 of folds, each containing an equal proportion of images from different categories and quality levels, ensuring that each fold was used once as a test set while the others served as the training set. We utilized the Receiver Operating Characteristic (ROC) curve to evaluate the sensitivity and specificity of LGAANet across different thresholds of classification.

## Results

### Experimental settings

We cropped blank areas of each image so that the width and height were equal and then scaled the cropped image to a resolution of 512 × 512. The eye-area mask was obtained through brightness and edge information, which was the alpha channel, denoted as MASK. The prediction model outputs a real value in the range of [0,1], outputs a 0/1 signal through the threshold judgment, and then compares it with the ground truth. In the experiment, the threshold (TH) was selected as 0.5.

### Color fundus photography dataset

The dataset annotations are listed in Table 2. For the color fundus photography (CFP) dataset, images with good I/C accounted for 61.0%, while GLU contained 86.5% of the poor I/C images. As for ‘blur’, the CFP dataset had 58.6% images without noticeable blur conditions, where DRIVE and NORMAL datasets had no blurry images. The same thing happened with regard to LC, and 68.3% of the images in the CFP dataset showed eligible contrast. In each aspect, images from LOCAL_1 and LOCAL_2 were inferior to those from DR_1 and DR_2.

**TABLE 2:** Dataset annotations.

Item	I/C		Blur		LC		Overall	
	**0**	**1**	**0**	**1**	**0**	**1**	**0**	**1**
LOCAL_1	158	41	94	105	85	114	142	57
LOCAL_2	78	25	59	44	41	62	77	26
DR_1	31	156	34	153	6	181	40	147
DR_2	36	199	120	115	78	157	117	118
GLU	45	7	48	4	42	10	50	2
NORMAL	2	24	0	26	0	26	0	26
DRIMDB	54	140	74	120	76	118	70	124
DRIVE	0	40	0	40	0	40	0	40
DR_UWF	215	285	163	337	50	450	168	332

Except for the DRIVE database, 80% of the CFP databases were randomly selected as the training set and 20% as the test set. We calculated the average prediction accuracy of the test set, attaining an acceptable result for the baseline; and with the addition of MASK, the accuracy increased to over 0.9. Spatial attention, multiscale, and self-attention algorithms all improved accuracy: BL+SelfAtt+MS achieved the best I/C and blur results, with accuracies of 0.947 and 0.924, respectively, and BL+SpatialAtt+MS produced the best results for LC, with an accuracy of 0.947.

Also, we added Gaussian white noise (Gauss) with a mean of 0 and a variance of 0.05 to images in the CFP datasets to improve the competence of the human visual system (HVS) -based algorithm. We conducted the experiments on each model, and the results showed robust properties, with the best accuracy over 0.85.

**FIGURE 4:**
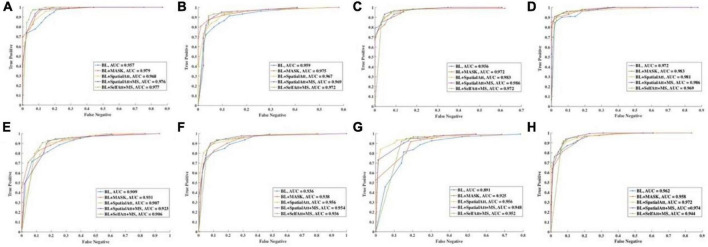
ROC curve of different items for **(A–D)** CFP datasets. **(E–H)** UWF datasets. **(A,E)** Detection of uneven illumination or color. **(B,F)** Detection of blur. **(C,G)** Detection of low contrast. **(D,H)** Overall quality.

ROC curves were drawn to further evaluate the performance of the models, as shown in Figure 4, and the areas under the ROC curves (AUCs) were calculated. For the CFP dataset, the AUC of each model on every item was over 0.95. Detailed information on accuracy and AUCs of the datasets is presented in Tables 3,4, respectively.

**TABLE 3:** Overall accuracy of different models on various datasets.

Model	CFP dataset				UWF dataset				Noise dataset			
	I/C	Blur	LC	Overall	I/C	Blur	LC	Overall	I/C	Blur	LC	Overall
BL	0.886	0.874	0.874	0.897	0.826	0.839	0.852	0.876	0.802	0.802	0.819	0.809
+MASK	0.922	0.902	0.917	0.919	0.852	0.862	0.889	0.893	0.819	0.822	0.839	0.826
+SpatialAtt	0.927	0.914	0.929	0.932	0.869	0.899	0.903	0.909	0.832	0.813	0.852	0.849
+SpatialAtt+MS	**0.947**	0.919	**0.947**	**0.944**	0.883	0.909	0.916	**0.926**	0.852	0.856	**0.879**	**0.873**
+SelfAtt+MS	**0.947**	**0.924**	0.942	0.939	**0.889**	**0.913**	**0.923**	0.923	**0.862**	**0.869**	0.873	0.869

The bold values in the table represent the highest values in the respective columns.

**TABLE 4:** The AUC of different models on various datasets.

Model	CFP dataset				UWF dataset				Noise dataset			
	I/C	Blur	LC	Overall	I/C	Blur	LC	Overall	I/C	Blur	LC	Overall
BL	0.957	0.959	0.956	0.972	0.909	0.936	0.891	0.962	0.862	0.879	0.874	0.884
+MASK	**0.979**	**0.975**	0.972	0.983	0.931	0.938	0.925	0.958	0.874	0.877	0.878	0.854
+SpatialAtt	0.968	0.967	0.983	0.981	0.907	**0.956**	**0.956**	0.972	0.888	0.899	0.89	0.922
+SpatialAtt+MS	0.976	0.969	**0.986**	**0.986**	**0.923**	0.954	0.948	**0.974**	0.891	**0.915**	**0.928**	**0.931**
+SelfAtt+MS	0.977	0.972	0.972	0.969	0.906	0.936	0.952	0.944	**0.905**	0.894	0.88	0.917

The bold values in the table represent the highest values in the respective columns.

**FIGURE 5:**
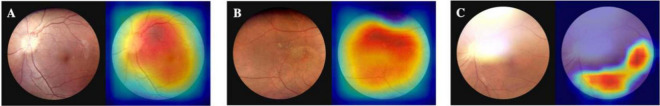
Heat map of the proposed model. **(A)** is a high-quality fundus image; the activated area is even and covers the whole image. **(B)** is a fundus image that contains a small area of uneven illumination, and therefore the top of the image is not activated. **(C)** contains a large area of strong light around the optic disk as well as the top of the image, and the rest area is properly activated.

Visualization of the prediction is interpreted by heat map, as shown in Figure 5. For high-quality images, the activated area is even and covers the whole image. When an image is suspected of poor quality, such as an area of uneven illumination, the model will not activate the designated area.

### Ultra-wide field fundus image dataset

In the UWF dataset, images with good quality accounted for 66.4%. Blurring was less common in UWF images, and the overall contrast was acceptable. The UWF dataset was not exploited for training, and we tested it with the proposed model as an external dataset. Performance on the BL was moderate, and compared with the BL, the following models all achieved better results. BL+SelfAtt+MS attained accuracies of 0.889, 0.913, and 0.923 for I/C, Blur, and LC separately.

The ROC curves for UWF images exhibited similar performance. BL+SpatialAtt+MS attained an AUC of 0.923 for I/C. Nevertheless, the AUCs for Blur and LC reached their maximums (both 0.956) in the BL+SpatialAtt model.

Table 5 provides a clear overview of the key technical terms and concepts used in the study, making it easier for readers from diverse backgrounds to understand the key aspects of the research.

**TABLE 5:** Appendix explains key technical terms and concepts.

Term and Concepts	Simple Explanation
Image Quality Assessment (IQA)	Evaluating how clear and useful an image is for medical purposes.
LGAANet	A smart system assessing eye images by analyzing both local details and the overall picture.
Multi-Source Heterogeneous Fundus (MSHF) Database	Collection of eye images from various sources and cameras.
Color Fundus Photography (CFP)	Standard color images of the retina.
Ultrawide-Field (UWF) Imaging	Wide-angle images capturing a broad view of the retina.
Attention Mechanisms	Focuses on significant parts of the image for analysis.
Saliency Maps	Highlights important image regions for decision-making in the neural network.
Multi-Level Spatially Pooled (MLSP)	Combines information from multiple levels of image analysis.
Global Average Pooling (GAP)	Computes the average of all feature maps in a neural network layer.
Spatial-Information-Retained (SIR)	Method preserving spatial details during image processing.
Receiver Operating Characteristic (ROC)	Graphical representation of a classifier’s performance.
Human Visual System (HVS)	System responsible for processing visual information in humans.
Areas Under the ROC Curves (AUCs)	Measure of the overall performance of a classifier.

## Discussion

In the realm of IQA, much of the existing literature has concentrated on singular modalities, predominantly CFP. The incorporation of alternative imaging modalities, such as portable fundus photography and UWF fundus imaging, which may be preferable in certain clinical scenarios, has been relatively overlooked. Wang et al represented a notable exception, employing both portable fundus camera images and public CFP datasets, demonstrating the machine learning model’s robust performance across these modalities (21).

To date, our research indicates a scarcity of research employing UWF images for fundus IQA, particularly studies that integrate CFP, portable fundus photography, and UWF imaging. Given that each imaging method addresses specific clinical requirements, developing an IQA system capable of accommodating this diversity is crucial. Furthermore, the challenge of ‘domain variance’ has been partially addressed in the prior research, which involved collecting images from both the source and target domains to train the network (22). Therefore, to fill these gaps, we compiled a multi-source heterogeneous fundus (MSHF) dataset, designed to meet the varied demands of clinical practice and mitigate the issue of domain variability.

Our Local and Global Attention Aggregated Deep Neural Network (LGAANet) was initially trained on images from portable and tabletop cameras, yet it demonstrated commendable adaptability and effectiveness when applied to UWF images. This underscores our model’s potential and versatility across different clinical settings. Previous contributions have introduced several notable networks, focusing on segmentation or generic evaluation, leveraging both conventional machine learning techniques and advanced deep learning methodologies. Our LGAANet, aimed at enhancing algorithmic performance and accommodating multi-source heterogeneous data, integrates both local and global information, resulting in incremental improvements in accuracy and AUC with each enhancement.

The advent of AI in clinical practice has underscored the importance of medical imaging quality assessment. Li et al. introduced DeepQuality, a deep learning-based system for assessing and enhancing the quality of infantile fundus images to mitigate misdiagnosis risks in infant retinopathy screening, demonstrating significant improvements in diagnostic models’ performance through analysis of over two million real-world images (23). This study introduces the innovative LGAANet for evaluating the quality of fundus images. Our MSHF dataset encompasses three primary types of retinal images: those captured by portable cameras, CFP images, and UWF images. These images were annotated by clinical ophthalmologists based on three distinct HVS characteristics and overall quality. The diversity of our dataset is visually represented through a spatial scatter plot. Developed on the sophisticated multi-level feature extractor SIR and incorporating an attention mechanism, the LGAANet was trained with images from portable cameras and CFP images. To evaluate the model’s robustness, we also tested it with UWF images and noisy data, analyzing overall accuracy and generating ROC curves to calculate the AUC for each set. Additionally, we propose the use of a salience map as a post hoc interpretability tool. This model paves the way for further exploration into AI-driven diagnostics, especially in the field of ophthalmology.

While the LGAANet has demonstrated significant advancements in fundus IQA, there are notable limitations that must be addressed in future research. One such limitation is the current model’s inability to enhance poor-quality images. Although LGAANet excels at assessing image quality, it does not yet possess the capability to improve subpar images to meet diagnostic standards. Future work should focus on developing algorithms that can transform low-quality images into high-quality ones, thereby increasing their diagnostic utility. Additionally, the reliance on a manually annotated dataset for model training and validation could introduce biases; thus, expanding the dataset and incorporating more diverse imaging conditions will be crucial for further validation. Finally, the generalizability of LGAANet to other imaging modalities and diseases outside of diabetic retinopathy and glaucoma remains to be explored. Addressing these limitations will be essential to fully realize the potential of LGAANet in clinical applications and to enhance the robustness and versatility of computer-aided diagnostic systems in ophthalmology.

## Data availability statement

The datasets presented in this study can be found in online repositories. The names of the repository/repositories and accession number(s) can be found in the article/supplementary material.

## Ethics statement

Ethical review and approval was not required for the study on human participants in accordance with the local legislation and institutional requirements. Written informed consent from the patients/participants was not required to participate in this study in accordance with the national legislation and the institutional requirements.

## Author contributions

SW: Conceptualization, Formal analysis, Investigation, Validation, Writing−original draft. WS: Formal analysis, Validation, Writing−original draft, Methodology. ZG: Formal analysis, Methodology, Validation, Data curation, Writing−original draft. XJ: Methodology, Writing−review and editing, Visualization. YW: Formal analysis, Validation, Writing−review and editing, Resources. YL: Writing−review and editing, Validation, Visualization. XM: Formal analysis, Methodology, Software, Validation, Visualization, Writing−review and editing. WW: Formal analysis, Methodology, Validation, Writing−review and editing. SX: Formal analysis, Methodology, Validation, Writing−review and editing. WR: Formal analysis, Methodology, Validation, Writing−review and editing. KJ: Conceptualization, Formal analysis, Resources, Supervision, Writing−review and editing. JY: Conceptualization, Funding acquisition, Resources, Writing−review and editing.
